# Systems approach in planetary health education for medical students: a mixed methods study

**DOI:** 10.1186/s12909-024-05341-1

**Published:** 2024-04-03

**Authors:** Rafaela Brugalli Zandavalli, Airton Tetelbom Stein, Tatiana Souza de Camargo

**Affiliations:** 1 Postgraduate Program in Science Education (PPgEci), Institute of Health Science, Federal University of Rio Grande Do Sul, Porto Alegre (ICBS-UFRGS), Primary Health Care Management - Conceição Hospital Group (GHC), Porto Alegre, Brazil; 2 Public Health Department, Federal University of Health Sciences of Porto Alegre (UFCSPA), Teaching and Research Management - Conceição Hospital Group (GHC), Porto Alegre, Brazil; 3 Postgraduate Program in Science Education (PPgEci), Institute of Health Science, Federal University of Rio Grande Do Sul, Porto Alegre (ICBS-UFRGS), Porto Alegre, Brazil

**Keywords:** Planetary health, Climate change, Education, Medical, Undergraduate

## Abstract

**Background:**

Introducing students to the "planetary health lenses" perspective is crucial. Comprehensive strategies for teaching this perspective are lacking, especially in the domains of "interconnection within nature (IWN)" and "systems thinking/complexity." There is also a scarcity of studies assessing medical students' opinions on planetary health and evaluating teaching strategies.

**Objective:**

To understand Brazilian medical students' perceptions and knowledge of planetary health (PH) and evaluate the application of the educational material "Patient and Clinic through the Lens of Planetary Health," which addresses "IWN" and "complexity" through the sociological lens of Actor-Network Theory, in an integrative course at a medical school in Brazil.

**Methods:**

A mixed-methods, quasi-experimental design involving two medical student classes during 2022/2023. Participants completed a questionnaire on sociodemographic data; pre- and post-intervention closed-ended questions about perceptions related to PH, and an open-ended questionnaire on experience and learning. Each student group presented a portfolio under the planetary health lenses regarding a real patient, developing a network diagram that described the social network involving both human and non-human actors with which this person is interconnected. The cohorts participated in "IWN" activities: a contemplative trail or reflection on belonging to the planet.

**Results:**

Ninety-six students and 9 professors participated. The majority of students (66.7%) reported significant or extremely significant learning from the sessions. There was an increase in perception of the need for physicians to incorporate PH into their clinical practice (*p* = 0.002; *r* = 0.46) and an intensification of the sense of interconnection with the environment (*p* = 0.003; *r* = 0.46). There was a gain in knowledge about how many diseases were related to PH (*p* < 0.02 for all 13 listed diseases). The majority (83%) found the sessions relevant or highly relevant and commented on their impact, both professionally and personally.

**Conclusions:**

Teaching PH in a medical school allowed students to learn from the patient's perspective, considering psychosocial and environmental determinants, about the intrinsic interdependence between population's health and PH. This strategy made a significant contribution by proposing pioneering didactics and offering valuable insights into the challenges and nuances of teaching PH.

**Supplementary Information:**

The online version contains supplementary material available at 10.1186/s12909-024-05341-1.

## Background

Anthropogenic changes are surpassing planetary boundaries within which humanity can thrive [1]. Consequently, climate change has emerged as the most significant global threat to public health in this century [2]. As a result of environmental degradation and the associated intensification of social inequalities, increases in chronic diseases, infectious diseases, and mental health issues are evident [3]. In this context, Planetary Health (PH) emerges as an expanding transdisciplinary field and social movement. It highlights the intrinsic interdependencies between human health and the health of Earth's ecosystems, with an overarching goal of global equity [4, 5].

Despite its paramount importance, the understanding and application of PH principles among healthcare professionals remain limited. Between 2019 and 2020, only approximately 15% and 11% of medical schools worldwide integrated climate change and air pollution, respectively, into their curricula [6]. A similar trend is evident in Brazil. A 2020 study revealed that 84.8% of healthcare professionals had not been exposed to practical or clinical content on planetary health during their training [7]. While there have been sporadic efforts to introduce PH in a handful of Brazilian universities [8], consistent inclusion of this theme in a Brazilian medical school curriculum only materialized in 2021 [9].

Several educational materials on PH have been developed globally to facilitate its introduction in universities [10]. However, these resources primarily emphasize the domain of "anthropocene and health" and occasionally addresses "movement building and systems change" [11]. Moreover, there's a noticeable scarcity of studies evaluating PH teaching strategies in undergraduate programs. The foundation of this study lies in the educational material titled "Patient and Clinic through the Lens of Planetary Health: Learning Guide for Health Education" [12]. This guide stands out due to its comprehensive coverage of all five domains recommended by the Planetary Health Educational Framework. These include “interconnection within nature (IWN)”, “anthropocene and health”, “systems thinking/complexity-based approaches”, “equity and social justice”, and “movement building and systems change” [3]. Particularly noteworthy is its emphasis on analyzing real patients and its pioneering approach to the domains of “interconnection within nature (IWN)” and “systems thinking/complexity-based approaches” in PH.

This article aims to glean insights into the perceptions and understanding of Brazilian medical students from a southern university regarding PH while evaluating the impact of implementing the educational guide "Patient and Clinic through the Lens of Planetary Health: Learning Guide for Health Education" in an integrative course of a Brazilian medical school.

## Methods

### Study design

This is a quasi-experimental study with a quali-quantitative methodology.

### Participants and survey

All professors and students of the Basic-Clinical Integration 3 course, within the third semester of the Medical School at the Federal University of *Rio Grande do Sul*, Brazil, during the months of August 2022 (class 1) and January 2023 (class 2), were conveniently invited. This course is part of the compulsory curriculum and seeks to integrate content from both basic and clinical courses, usually allowing students to engage in interviews with patients. Subsequently, students compile a portfolio detailing the disease and the patient interviewed. Students who did not respond to the informed consent form or declined to participate in the study were excluded. Participants who did not adequately respond to either qualitative or quantitative evaluations were also excluded.

The instrument consisted of sociodemographic data; closed questions about PH (adapted from the Brazilian Pilot Massive Open Online Course in Planetary Health Education [7]); and an open questionnaire with 9 questions regarding the experience and learning. For some questions related to PH, a Likert scale structure ranging from 1 to 5 was used, with 5 representing the highest response values. The instrument (in Portuguese and English) can be found in Supplementary Material 1.

### Intervention

The educational intervention consisted of a module of lessons centered on the theme of PH within the Basic-Clinical Integration 3 course during the third semester at the Medical School of the Federal University of *Rio Grande do Sul* (Table 1). Each class was divided into small groups (1 professor for approximately 8 students), each tasked with interviewing a patient admitted to the university hospital and developing a portfolio [13] with questions about their clinical case through the lens of PH, covering areas such as epidemiology, basic sciences, clinical, and even inquiries about which individual, community, and health system approaches could be proposed. Generally, the second group of students received a slightly different intervention than the first: an additional lecture was included, and the "IWN" activity was modified.

**Table 1:** Overview of the lesson plan

August 2022 (1st class of students)	Pre Class Material	Class 1 (2 ½ hours)	Group Assignment	Class 2 (2 ½ hours)
	Short thought-provo-king text and videos handed out for students to read before the first class	Patient Interview + IWN activity (activity 1: Trail)	Development of the portfolio as a home activity (delivery in text and presentation form)	Portfolio Presentation and Discussions (PH researchers were invited)
January 2023 (2nd class of students)	Class 1 (2 ½ hours)	Class 2 (2 ½ hours)	Group Assignment	Class 3 (2 ½ hours)
	1 h 16 min pre-recorded lecture about PH (available in Portuguese: https://youtu.be/Uqs1SLDyj8E)	Patient Interview + IWN Activity (activity 2: Photographs)	Development of the portfolio as a home activity (delivery in text and presentation form)	Portfolio Presentation and Discussions (PH researchers were invited)

The final item of this portfolio was the task of drafting a network diagram, placing the patient at the center, and mapping the connections between the various actors that appeared in the case (individual, family, psychosocial, environmental aspects, and PH keywords such as air pollution, deforestation/burning, climate change, heatwaves, natural disasters, equity or inequity, migrations, water/food insecurity, mental health, and infectious diseases). This process created networks, illustrating the complexity of factors present in a society, which includes the influence of non-human actors in its construction.

The "IWN" "Trail" activity (Class 1) consisted of a walk through the university's garden, located in an urban area, where students encountered posters prompting reflections on the subject. The "IWN" activity for Class 2 involved reading a support text followed by each student sharing with their small group a photo and/or account of a moment when they felt part of the planet. All activities are detailed in the published educational materials [13].

These teaching approaches for "IWN" and "systems thinking/complexity" were developed based on the theoretical foundation of Actor-Network Theory, a sociological theory that discusses the humanity/nature division and considers both humans and non-humans as acting within networks and contributing to the construction of society [14].

### Data analysis

The qualitative data underwent Bardin's content analysis (using NVIVO 1.5 software). For the quantitative data (analyzed using Python), the Likert scale data were considered continuous, and opinions on the 16 different diseases, classified as associated or not with PH, were evaluated categorically. Outcome variables were assessed before and after the intervention, and the Wilcoxon test was conducted. The biserial correlation (r) was calculated for each relationship.

Subgroup analyses were conducted based on age, gender, ethnicity, family income, and class. Additional analyses were carried out with the group of students who scored 1, 2, or 3 on the Likert scale in the pre-test PH questions, examining their learning differences compared to the group that initially scored 4 or 5 on the scale. The Student's T-tests (for continuous data) and Fisher's tests (for categorical data) were used. A *p*-value of < 0.05 was considered statistically significant for all tests.

### Ethical aspects

All participants completed the informed consent form, and the study was approved by the Ethical and Research Committee of the Federal University of *Rio Grande do Sul*.

## Results

Of the 110 students invited, 8 declined to participate (class 1 *n* = 7; class 2 *n* = 1), and 6 students (class 1 *n* = 1; class 2 *n* = 6) were disqualified for not responding to the informed consent form or the post-test. Ninety-six students (class 1 *n* = 43; class 2 *n* = 53) and nine professors were included in the qualitative analyses. Only 90 students (class 1 *n* = 43; class 2 *n* = 47) were enlisted for the quantitative analyses, as six students did not complete the quantitative pre-test.

### Quantitative results

The majority of the students were female (53.12%), white (68.75%), with a family income of 5–10 minimum wages (36.46%), and had not heard of PH before (58.33%); average age ± standard deviation (24.41 ± 5.8). Students who had some prior knowledge of PH from a lesson indicated, in response to an open-ended question, that this lesson took place in another undergraduate course in medicine or veterinary science and was merely a brief mention of the topic (Table 2).

**Table 2:** Sociodemographic variables and previous knowledge about planetary health

Variable	Data^a^	
Age		
Mean ± standard deviation	24.41 ± 5.8	
Minimum, maximum	19. 56	
Gender		
Female	51	53.12%
Male	44	45.83%
Other (Female or Male Transgender, Non-binary, others)	1	1.04%
Ethnicity		
White	66	68.75%
Brown	21	21.88%
Black	6	6.25%
Yellow or indigenous	2	2.08%
Non-respondents	1	1.04%
Family Income (in minimum wages)		
1–4	33	34.38%
5–10	35	36.46%
11 or more	27	28.13%
Non-respondents	1	1.04%
Brazilian Birth Region		
South	52	54.17%
Southeast	23	23.95%
North East	7	7.29%
Midwest	5	5.21%
North	3	3.13%
Non-respondents	6	6.25%
Before this class, have you heard of "Planetary Health"?		
No	56	58.33%
Yes	31	32.29%
I’m unsure	9	9.38%
If yes, how did you have the first contact with the theme? (it's possible to mark more than 1 option)		
Internet	17	55,%
Class	13	42%
Social Network	10	32%
Friends/Family	7	23%
Television Program	5	16%
Scientific Article	4	13%
Podcast	2	6%
Lecture	2	6%
TelessaúdeRS-UFRGS Planetary Health Open Online Course	1	3%

^a^Numeric data presented as means ± standard deviation and categorical data as their frequency

**Fig. 1:**
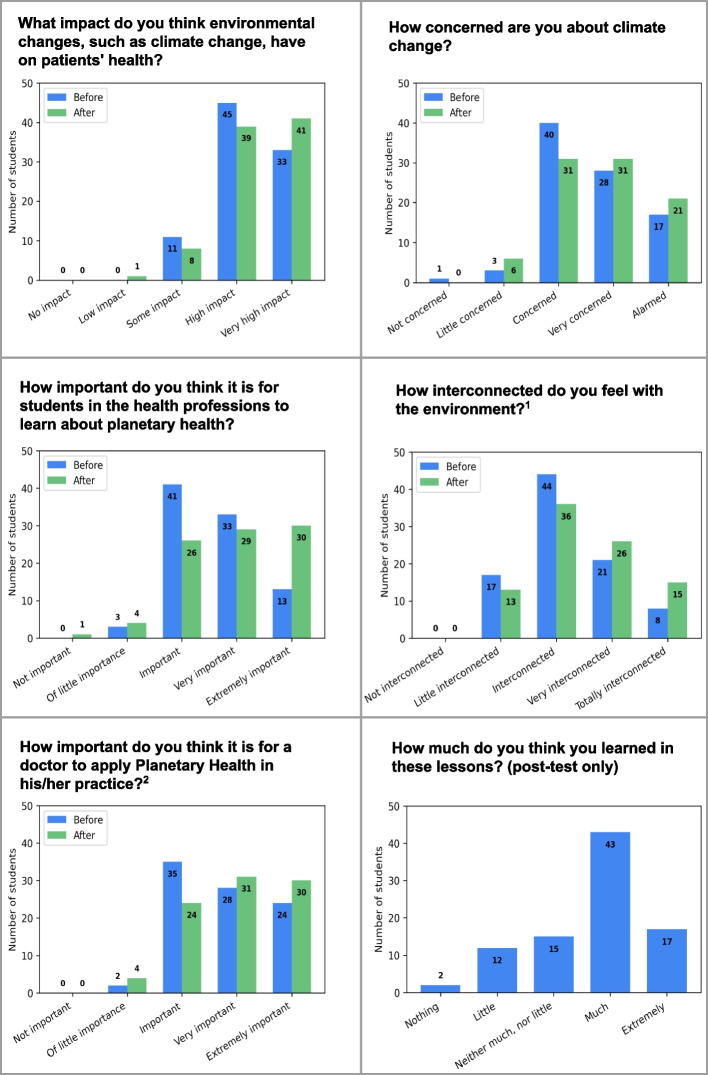
Pre and post-evaluation of perceptions about planetary health (*n* = 90). ^1^ *p* = 0.003; *r* = 0.46. ^2^ *p* = 0.002; *r* = 0.46

**Fig. 2:**
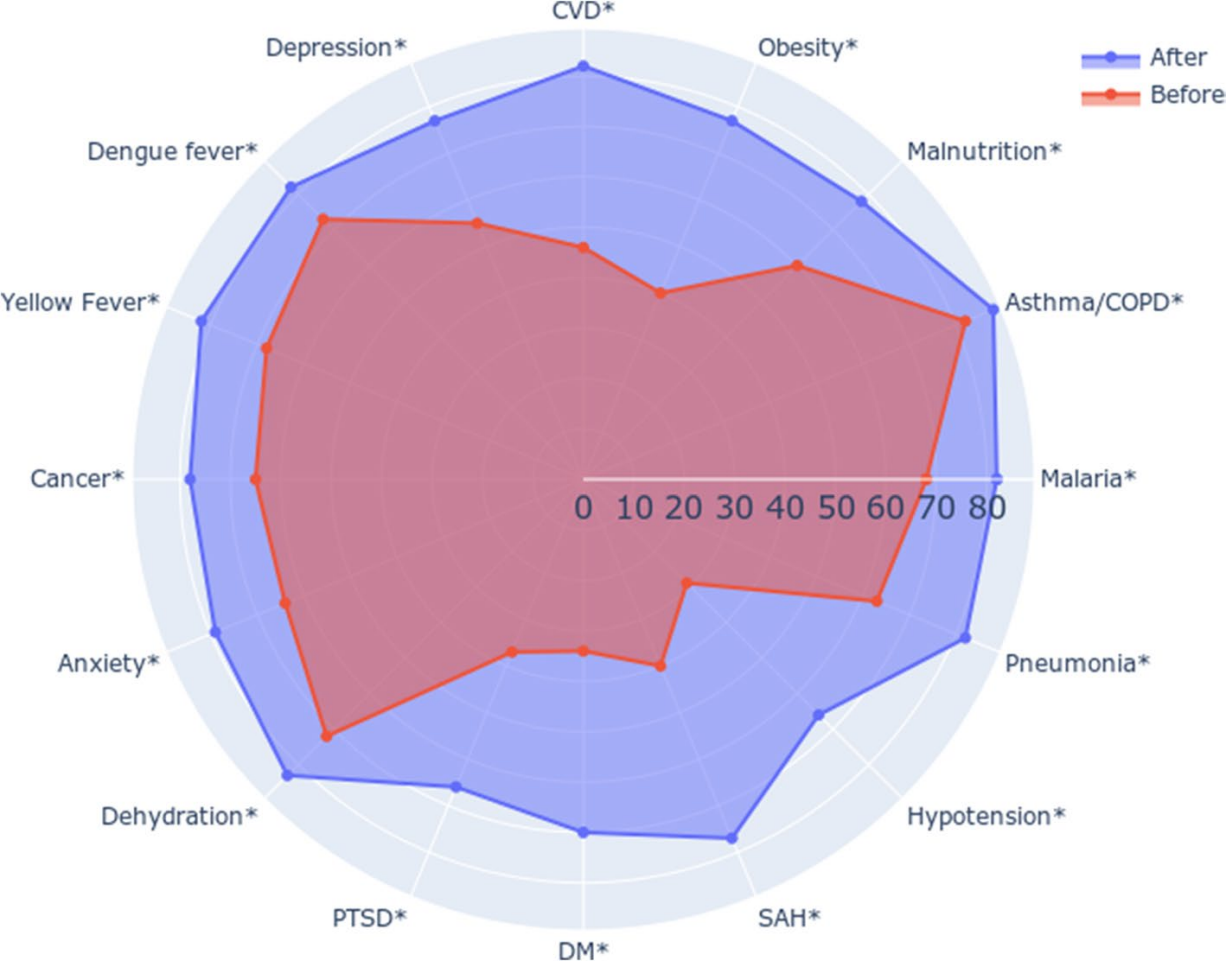
Knowledge about diseases related to environmental changes (*n* = 89). CVD Cardiovascular diseases (stroke and acute myocardial infarction), COPD Chronic obstructive pulmonary disease, SAH Systemic arterial hypertension, DM Diabetes mellitus, PTSD Post-traumatic stress disorder. * *p* < 0.05

In the pre- and post-test analyses of the students, there was a significant improvement in the sense of interconnection with the environment, and the importance of physicians applying PH in their practice. Perceptions about the impact of climate change on patient health, concerns about climate change, and the importance of the topic for the health field did not show significant gains after the intervention (Table S1 (Supplementary Material 2); Fig. 1). However, a secondary analysis, assessing changes in these areas solely with students in the 1st quartile, revealed statistically significant gains in all these domains (*p* < 0.01 for all domains). The majority felt they learned a lot from the educational sessions (Table S1 (Supplementary Material 2); Fig. 1). The students significantly enhanced their understanding of the correlation of all diseases with environmental changes (Table S2 (Supplementary Material 2); Fig. 2).

There was no statistically significant difference when analyzing each of the questions about perceptions of PH and knowledge of diseases related to PH in general when comparing the subgroups of the first class to the second class. However, in the latter, students more specifically learned that cardiovascular diseases (stroke and acute myocardial infarction) and depression were related to PH (*p* < 0.05). There were also no significant differences in learning among other subgroups (age, gender, ethnicity, and family income), which were analyzed to determine if these demographic factors influenced outcomes.

The graphs (Fig. 1) display the number of students who chose each option before (in blue) and after (in green) the intervention. Comparing the average responses before and after, there was a statistically significant increase in the sense of interconnection with the environment (^1^ *p* = 0.003; *r* = 0.46) and in the perception of the importance for physicians to apply PH in their practice (^2^ *p* = 0.002; *r* = 0.46). The detailed table corresponding to this figure can be found in Table S1 (Supplementary Material 2).

The graph (Fig. 2) shows the number of students who identified each disease as related to PH in response to the question: “Which of the following health problems in practice are associated with environmental changes, such as climate change? (select as many as you think appropriate)”. The count before the intervention corresponds to the shape closer to the center of the figure and is in red; the count after the intervention is in blue. The detailed table corresponding to this figure is shown in Table S2 (Supplementary Material 2). There was a significant increase (*P* < 0.05) in the number of students who understood the association of each disease as related to PH for all conditions.

### Qualitative results

#### Students' perceptions

The general perception of the students regarding the relevance of the PH sessions was that the majority (83%) found them relevant or highly relevant (Table 3). The methodology was praised for its active participation, creativity, interactivity, the proposal to interview a real patient and reflect on PH from their perspective, the quality and accessibility of the provided study references, and the discussion of clinical cases associated with group portfolio presentations (Table S3 (Supplementary Material 2)). The few who rated the relevance poorly (13.3%) (Table 3) typically justified this due to methodological reasons related to the playfulness of the intervention, the perception that the approach was either too dense/heavy or superficial, and the format of the lecture (both recorded and in-person) (Table S3 (Supplementary Material 2)).

**Table 3:** General perception on the relevance of the PH sessions “Did you find these PH sessions relevant? How much so?”

Category	Class 1 + 2 n (%)	Class 1 n (%)	Class 2 n (%)	Examples
Relevant or highly relevant	75 (83%)	37 (86%)	38 (80%)	“I consider it of utmost importance for the current moment." and "Yes, I find it very relevant to address such topics because we live with climate change and, therefore, with its consequences in various aspects of our lives, such as health.”
Comments on Knowledge Gain	18	8	10	“(…) "greatly enhancing the potential for academic and human development of the students." and "I was able to see the extent of the impact of planetary health on patients' lives."
Comments on Innovation and Surprise	7	4	3	“The project is very cool and innovative! It's a subject I wouldn't have known about otherwise.", "It plays an important role in introducing us to a medical and citizen approach that hadn't been explored before.”
Praises	5	0	5	“I must commend the thinkers behind this project(…)!", and "It was executed perfectly.”
Comments on Gratitude	3	3	0	“Thank you for the learning opportunity and the experience.”
Slightly or Moderately Relevant	12 (13.3%)	4 (9.3%)	8 (17%)	Justifications: 4 for methodological reasons, 4 without explanation, 2 thinking that the topic isn't very relevant in medicine, 1 for already being familiar with the topic, and 1 for reporting little learning plus methodological critique: "[PH] should only be investigated in patients due to high exposure to factors, without the need to ask much about it.", "(…) I believe that most of these discussions belong to other areas of knowledge" and "Concern for the environment is a much more constant agenda for 21st-century youth than for those born in the twentieth century. In this sense, presenting global health as an important aspect seems more like a recollection of what has already been studied than a new perspective."
Not relevant	3 (3.3%)	0 (0%)	3 (6.3%)	Student justifications related to methodological aspects

#### Personal impacts

In terms of personal impacts, students reported being more aware of the subject after the intervention, rethinking their habits, and, to a lesser extent, seeking to act collectively for system change. It is noteworthy that one student highlighted the benefit of the healthy and respectful debate that the tasks facilitated, a characteristic so vital for contemporary societal life. Other students emphasized that they will cherish the well-being they experience when in contact with green/blue environments. However, one student reported anxiety related to the topic (Table S4 (Supplementary Material 2)).

#### Professional impacts

Regarding the impact on the students' professional lives, we observed that they reported an expanded view of the health-disease process. This allowed them to guide individuals about the co-benefits of health and the environment and the change in habits. They felt compelled to learn more about PH, recognized their role in reducing the environmental footprint of the healthcare system, and, to a lesser extent, saw their potential to contribute to broader systemic changes in society (Table S5 (Supplementary Material 2)).

#### Timing and module structure

The timing of the PH lessons was considered by several students as not ideal due to the overlap with demands from other courses combined with the requirements of PH activities. Students' suggestions ranged from holding the sessions at the beginning of the semester, in different semesters, or making better use of class time, such as setting aside a week for project development without in-person classes. Suggestions to cover this topic in other courses, or to create separate elective or mandatory courses, were also mentioned (see Supplementary Material 2).

## Discussion

The results indicated improvements in various aspects related to the students' knowledge of PH, both in their subjective perception (sense of learning from the sessions; sense of interconnection with the environment and the importance of its practical application) and when objectively measuring how many diseases they could relate to PH. The perception of the impact that environmental changes, such as climate change, have on patients' health; concern about climate change; and the importance for students in health professions of learning about PH did not show significant changes, possibly due to their high prior concern. The secondary analysis, which showed that students whose responses were in the first quartile experienced statistically significant gains in all items, supports this hypothesis. It indicates that those with the least awareness of the connections between environmental changes and health were able to significantly enhance this understanding through the educational intervention.

The qualitative data indicate that, in general, students recognized the relevance of these educational sessions because they addressed this very current and pertinent topic for life in society and expanded one's perspective when facing a patient. Some students displayed limited openness to what was being introduced for teaching, citing that environmental education was already imparted during their school years or seeing no connection with medicine. However, although environmental education and traditional sustainability approaches have been on the rise in both formal and informal educational settings in recent years, PH introduces distinct novel elements. PH emphasizes the connection to human health, addresses climate changes, and adopts a systemic viewpoint on the complexity of relationships, also integrating the issue of inequity.

The innovative methodology was appreciated by the majority of students, but the multitude of diverse methods in a brief period made it dense. Both classes sought deeper exploration and more time to understand the content. Despite the availability of a wide range of materials, from quick multimedia to in-depth articles, they found it challenging to fully engage with these options due to time constraints. Even though the classes had slightly different instructional approaches, both were effective.

The close-ended question, "How interconnected do you feel with the environment?", although in some ways alluding to the unreal division between humanity and nature, showed a statistically significant increase after the intervention. This indicates that the "IWN" activities contributed to feelings of interconnection. However, we did not explore to what extent this reflects a commitment to environmental protection, as another study did with healthcare professionals, where almost half expressed a commitment to protection [7].

Concerning their personal lives, students indicated that the approach effectively stimulated more thoughtful reflections on their habits, with some considering more collective actions. One student expressed that such lessons can add to students' anxiety about environmental degradation, termed eco-anxiety, something that should be anticipated and addressed when discussing this topic. Some students conveyed feelings of helplessness; others felt a sense of urgency and a call to action to address these issues. However, in general, the educational intervention did not seem to significantly alter the level of concern about climate change, which was already high before the sessions.

Eco-anxiety is a reasonable and functional response and should be channeled to generate momentum, rather than paralysis or mental health pathologies [15]. It is essential to highlight positive experiences and emphasize that every action now, in any field of knowledge and potential intervention, matters, driving collective movement and fostering hope [16]. This should be done without losing our ability to appreciate beauty and to approach transformative tasks at a non-debilitating pace [17]. Perhaps one way to understand the Anthropocene is to treat it as an immense rite of passage, which, while painful and unfair, will result in mature adults accountable for their actions [18].

Regarding the impact on students' professional lives, there was a significant increase after the intervention. Numerous comments were made about the broadened perspective on the health-disease process, which offers the potential to contemplate new guidance for protection against risk factors and in favor of co-benefits. This also includes reducing the environmental footprint of the healthcare system and making broader, scientifically-backed health recommendations in society. Compared to another study, many healthcare professionals responded affirmatively about the total or partial applicability of PH in their field and very few felt it was not applicable [7]. To truly assess the personal and professional impacts, this study has limited evaluative capacity, as a prolonged period of observation would be required to gauge the actual repercussions.

One teacher noted that the students did not see themselves as active protagonists for PH, suggesting the addition of a question to the portfolio: “In what actions could you, as a student, engage to promote the health of individuals, such as your interviewed patient?”. In subsequent sessions with a following class, there was also a suggestion that the students develop an intervention project based on the studied case. Regarding this research's assessment, we recognized that we could have evaluated whether the students felt encouraged to initiate change while still in their student roles. To our knowledge, one student mentioned considering the establishment of a University Planetary Health Club or League.

We consider it crucial to situate this educational intervention within the context of Brazil's social and health system realities. It is vital to reflect upon the social issues addressed by PH, encompassing the domain of “equity and social justice” as outlined in the PH Education Framework. Particularly in Brazil, where students are daily witnesses to social inequality, understanding how PH interacts within this context and affects people's lives is imperative. There is a significant overlap between social and environmental health determinants, as both are rooted in historical structures of domination and also mutually influence each other. For instance, deteriorating environmental conditions exacerbate social inequalities, leading to increased food insecurity, restricted access to safe housing prompting population migrations and increased violence, and greater suffering for those in manual labor on hot days. Similarly, social conditions are linked to environmental determinants, as, for example, lower-income populations may live in areas with limited access to green spaces and higher pollution levels [3].

Reflecting on social and environmental justice, it becomes evident that those who suffer the most from environmental degradation are often those who contribute the least to it. Therefore, recommendations for safeguarding human and planetary health must be contextualized, taking into account social justice and equity promotion [3]. Health practice within the Brazilian Health System (SUS) needs to acknowledge this reality, recognizing that environmental degradation places an even greater burden on the SUS, from primary health care (PHC) to tertiary care. Strategies for protecting individuals and mitigating environmental determinants that adversely affect health are imperative. PHC plays a pivotal role in surveillance policies and promoting health, as it is closely connected to the communities where people live, allowing for the promotion of health education and social mobilization towards healthier and safer community environments. These important discussions were woven throughout our educational activities.

It is also important to reflect on how the current medical education process has influenced students' learning, including their receptiveness to the topic and the didactic format used, which incorporated reflective and artistic elements under a patient-centered perspective, coupled with theoretical aspects featuring interdisciplinary themes. While the Flexnerian model has greatly contributed to medical education, its emphasis on the biomedical approach, focused on disease, hospitals, and medical specialties, has led medical educational programs to adopt a reductionist view of health, leaving little room to consider the social, psychological, and environmental dimensions of health, as well as the broad spectrum of health that goes beyond medicine and its practitioners [19, 20]. The holistic approach we implemented was well-received by many students, but faced resistance from some. This underscores the necessity for greater appreciation within educational systems, among medical teachers, and within society at large for topics related to public health and multidisciplinary work [19, 20].

Currently, according to the Brazilian National Curricular Guidelines, there is also a recommendation that specific themes, such as those related to gender, environment, and ethnic-racial issues, permeate the entire medical education [21]. Consistent with global recommendations of introducing medical students to a perspective termed as "planetary health lenses" [22–25], some entities also propose the transversal integration of PH content throughout the course [6, 26]. This would significantly contribute to the students' understanding, as their comprehension of any subject would be viewed from the PH perspective. There is a pressing need to take on the challenge of coordinating such an implementation, possibly coupled with the need to train various professors on the topic. However, we believe it is crucial that PH is addressed comprehensively in some course—be it integrative, public health, clinical, or solely focused on PH. This approach ensures that students envision the patient as a whole [27] and grasp PH’s full scope and its complexity [28], as was done in this activity. Otherwise, certain PH educational holistic domains, such as “IWN” and “systems thinking/complexity”, run a significant risk of not being addressed throughout the course.

The intervention was generally very significant within the available class time and fulfilled its role of introducing the subject and providing a comprehensive approach to it. Additionally, longer or periodic activities throughout the course may provide a broader understanding of all domains, especially these two domains, allowing more time for content assimilation, exploration, and debate on its nuances and complexities [29]. They require philosophical reflections [30] and practice in “connecting the dots”. Moreover, with more time, students could become familiar with more examples of creating momentum and system change, enabling them to see themselves as protagonists and envision creative possibilities for intervention. Planetary Health (PH) addresses issues of knowledge and skills involving cognitive, social, behavioral, and affective processes [31, 32]. The journey towards mastery in this new area of expertise unfolds gradually along the learning curve, involving a process of refinement and maturation [32].

This study contributes to the field of planetary health education, as it qualitatively and quantitatively assessed the first known Brazilian didactic materials on planetary health and elaborates on the challenges and nuances of teaching planetary health to medical students. As a result of the sessions, it is also worth noting that the course teachers adopted permanently this planetary health module and included a question for all interviews/portfolios conducted throughout the course during the whole semester: "Relate aspects of the interviewed patient and the studied disease to Planetary Health.".

The limitations of this study are that it was conducted at only one university and there isn't a validated objective questionnaire about learning in planetary health available in the scientific literature. However, this study adds to the literature by reflecting on the successes and challenges related to the utilized questionnaire, which will serve as a basis for the development of enhanced ones in the future. Future educational interventions on planetary health should consider allocating more time for teaching this subject to students, focusing on listening to and dialoguing more with students, exploring the domain of "movement building and system change" more thoroughly, and considering interviewing real patients in a Primary Health Care (PHC) context, where the "minute for the planet" individual approach [33] and community approaches can be explored more closely to their territory. Future studies should also consider assessing how students perceive themselves as agents of change in their current role as students.

## Conclusions

This study evaluated the implementation of PH teaching strategies in a Brazilian medical school, marking one of the first experiences of its kind in the country. Upon conducting a scoping review, it is also evident that this is among the few global initiatives assessing an educational strategy in undergraduate medical curricula. This research evaluates the use of the first Brazilian educational material on PH for undergraduate studies, which has made a significant contribution to the international PH teaching methodology. This is especially true concerning the pioneering approach to teaching the domains of "IWN" and “systems/complexity thinking”, enabling students to "connect the dots" between real patients and PH.

The results indicated improvements in students' knowledge regarding PH, both in terms of subjective perception (sense of interconnection with the environment, importance for practical application) and in objective measures of how many diseases they could associate with PH. Qualitative data underscored the relevance of the educational sessions, praising the active participation methodology, creativity, and interactivity of the activities. However, some students displayed limited receptiveness to the proposed teachings and there were suggestions for enhancements, such as the need for deeper content exploration in a participatory manner and encouraging student leadership in PH. The authors also encourage future educational approaches to enable students to experience Planetary Health initiatives engaging more closely with the population and community within the PHC setting.

In conclusion, this study offers valuable insights into the challenges and nuances of teaching PH to medical students, contributing to the United Nations (UN) 2030 Agenda for Sustainable Development [34]. The instruction of PH is essential for future physicians to understand the intricate interconnection between human health and the planet's well-being and to act in various personal and professional realms towards inhabiting this planet in a manner that's more equitable for all beings.

### Supplementary Information

**Supplementary Material 1.**

**Supplementary Material 2.**

## Data Availability

The datasets used and/or analysed during the current study are available from the corresponding author on reasonable request.

## Abbreviations

PH: Planetary Health
IWN: Interconnection within Nature
UFRGS: Federal University of Rio Grande do Sul
