# Supplementary material for: Systems approach in planetary health education for medical students: a mixed methods study
Source: BMC Med Educ. 2024 Apr 3;24:365. doi: 10.1186/s12909-024-05341-1 (PMC10988787; doi:10.1186/s12909-024-05341-1)
Supplement: Supplementary file 1 — Supplementary Material 1. [file 12909_2024_5341_MOESM1_ESM.pdf]

Supplementary Material 1 - Instruments

INSTRUMENTS

ANNEX 1- Objective Questionnaire (English version)

ANNEX 2 - Questionnaire with Semi-structured Questions and Dissertation Answers (written) (English version)

ANNEX 3 - Questionário Objetivo (Portuguese version)

ANNEX 4 - Questionário com Perguntas Semiestruturadas e Respostas Dissertativas (por escrito) (Portuguese version)

ANNEX 1- Objective Questionnaire (English version)

Sociodemographic Data - Pre-Test Only

1. Age:____

2 . Gender:

- Female
- Male
- Other (Transgender female, Transgender male, Non-binary, other). If possible, specify:______________

3. Place of Birth (City/State):_________________

4. Race/ethnicity:

- Black
- Brown
- Yellow or indigenous
- White

5. Family income (in minimum wages):

- 1-2
- 3-4
- 5-6
- 7-8
- 9-10
- 10-12
- 13-14

- 15 or more

Planetary Health - Pre-Test Only

1. Have you heard of the term "planetary health"?

- Yes.
- No
- I am uncertain

2. If yes, how did you first get in touch with the topic?

- Internet
- Class
- Podcast
- TV program
- Scientific article
- Social networks
- Lecture
- By friends/family
- Outro (specify)_______________

3. If you answered "Class" in the previous question, please specify which class of which institution or course you had the first contact with the theme of Planetary Health.

____

Planetary Health - Pre- and Post-Test

1. What impact do you think environmental changes, such as climate change, have on patients' health?

- No impact
- Low impact
- Some impact
- High impact
- Very high impact

2. How concerned are you about climate change?

- 1 Not concerned
- 2 Slightly concerned
- 3 Concerned
- 4 Very concerned
- 5 Alarmed

3. How important do you think it is for students in the health professions to learn about planetary health?

- 1 Not at all important
- 2 Not very important
- 3 Important
- 4 Very important
- 5 Extremely important

4. Which of the following health problems in practice are associated with environmental changes, such as climate change? (select as many as you think appropriate)

( ) Malaria ( ) Asthma/Chronic Obstructive Pulmonary Disease ( ) Malnutrition ( ) Obesity

( ) Cardiovascular disease (stroke and acute myocardial infarction) ( ) Depression

( ) Dengue ( ) Yellow Fever ( ) Cancer ( ) Anxiety ( ) Dehydration

( ) Post-traumatic stress disorder ( ) Diabetes Mellitus

( ) Systemic Arterial Hypertension ( ) Hypotension ( ) Pneumonia

5. How interconnected do you feel with the environment?

- 1 Not at all interconnected
- 2 Not very interconnected
- 3 Interconnected
- 4 Very interconnected
- 5 Fully interconnected

6. How important do you think it is for a doctor to apply Planetary Health in his/her practice?

- 1 Not at all important
- 2 Not very important
- 3 Important
- 4 Very important
- 5 Extremely important

7. How much do you think you learned in these lessons? (post-test only)

- 1 Not at all
- 2 Not much
- 3 Not a little, not too much
- 4 Very much
- 5 Extremely

ANNEX 2 - Questionnaire with Semi-structured Questions and Dissertation Answers (written) (English version)

Planetary Health - Post-test only

- Did you find these SP meetings relevant? How relevant?

- What did you think of the teaching methodology used?

- How can the meetings on planetary health help you in your professional practice?

- How can the meetings on planetary health help you in your personal life?

- Your observations about these planetary health meetings and suggestions to improve it:

ANNEX 3 - Questionário Objetivo (Portuguese version)

Dados Sociodemográficos - Somente Pré Teste

1. Idade:____

2 . Gênero:

- Feminino
- Masculino
- Outros (Feminino transgênero, Masculino transgênero, Não-binário, outros). Se possível, especificar:______________

3. Naturalidade (cidade/Estado):_________________

4. Cor:

- Preta
- Parda
- Amarela ou indígena
- Branca

5. Renda familiar (em salários mínimos):

- 1-2
- 3-4
- 5-6
- 7-8
- 9-10
- 10-12
- 13-14
- 15 ou mais

Saúde Planetária - Somente Pré Teste

1. Você já ouviu falar do termo “saúde planetária”?

- Sim.
- Não
- Tenho incerteza

2. Se sim, como você teve o primeiro contato com o tema?

- Internet
- Aula
- Podcast
- Programa em TV
- Artigo científico
- Redes sociais
- Palestra
- Por amigos/familiares
- Outro (Especifique)______________

3. Se você respondeu "Aula" na pergunta anterior, especifique que aula de que instituição ou curso você teve o primeiro contato com o tema da Saúde Planetária.

____

Saúde Planetária - Pré e Pós Teste

1. Qual o impacto que você acha que as mudanças ambientais, como mudanças climáticas, têm sobre a saúde dos pacientes?

- Sem impacto
- Baixo impacto
- Algum impacto
- Alto impacto
- Muito alto impacto

2. Qual é o seu nível de preocupação acerca das mudanças climáticas?

- 1 Não preocupado/a
- 2 Pouco preocupado/a
- 3 Preocupado/a
- 4 Muito preocupado/a
- 5 Alarmado/a

3. Quão importante você acha que é para os estudantes das profissões da saúde aprenderem sobre saúde planetária?

- 1 Nada importante
- 2 Pouco importante
- 3 Importante
- 4 Muito importante
- 5 Extremamente importante

4. Quais dos seguintes problemas de saúde na prática se associam às mudanças ambientais, como às mudanças climáticas? (selecionar quantas achar conveniente)

( ) Malária ( ) Asma/Doença Pulmonar Obstrutiva Crônica ( ) Desnutrição ( ) Obesidade

( ) Doença cardiovascular (infarto cerebral e cardíaco) ( ) Depressão

( ) Dengue ( ) Febre amarela ( ) Câncer ( ) Ansiedade ( ) Desidratação

( ) Transtorno de estresse pós traumático ( ) Diabetes Mellitus

( ) Hipertensão Arterial Sistêmica ( ) Hipotensão ( ) Pneumonia

5. O quão interconectado você se sente com o meio ambiente?

- 1 Nada interconectado
- 2 Pouco interconectado
- 3 Interconectado
- 4 Muito interconectado
- 5 Totalmente interconectado

6. O quanto você considera importante o médico aplicar a Saúde Planetária em sua prática?

- 1 Nada importante
- 2 Pouco importante
- 3 Importante
- 4 Muito importante
- 5 Extremamente importante

7. O quanto você acha que aprendeu nessas aulas? (somente pós-teste)

- 1 Nada
- 2 Pouco
- 3 Nem pouco, nem muito
- 4 Muito
- 5 Extremamente

ANNEX 4 - Questionário com Perguntas Semiestruturadas e Respostas Dissertativas (por escrito) (Portuguese version)

Saúde Planetária - Somente Pós Teste

- Você considerou estes encontros sobre SP relevantes? Quanto?

- O que você achou da metodologia de ensino utilizada?

- Como os encontros sobre saúde planetária podem te ajudar na sua prática profissional?

- Como os encontros sobre saúde planetária podem te ajudar na sua vida pessoal?

- Suas observações sobre estes encontros sobre saúde planetária e sugestões para aprimorá-lo:
