# Supplementary material for: Systems approach in planetary health education for medical students: a mixed methods study
Source: BMC Med Educ. 2024 Apr 3;24:365. doi: 10.1186/s12909-024-05341-1 (PMC10988787; doi:10.1186/s12909-024-05341-1)
Supplement: Supplementary file 2 — Supplementary Material 2. [file 12909_2024_5341_MOESM2_ESM.pdf]

Supplementary Material 2 - Extra Quali-Quantitative Analysis.

Table S1. Pre and post-evaluation of perceptions about planetary health (n=90).

Question	Answers	Pre-Test		Post-Test		p	r
		N	%	N	%		
What impact do you think environmental changes, such as climate change, have on patients' health?	No impact	-	-	-	-		
	Low impact	-	-	1	1.1%		
	Some impact	11	12.2%	8	8.9%		
	High impact	45	50.0%	39	43.3%		
	Very high impact	33	36.7%	41	45.6%		
	Non-respondents	1	1.1%	1	1.1%		
	**Mean ± SD**	4.25 ± 0.66		4.35 ± 0.69		0.096	0.21
How concerned are you about climate change?	Not concerned	1	1.1%	-	-		
	Little concerned	3	3.3%	6	6.7%		
	Concerned	40	44.4%	31	34.4%		
	Very concerned	28	31.1%	31	34.4%		
	Alarmed	17	18.9%	21	23.3%		
	Non-respondents	1	1.1%	1	1.1%		
	**Mean ± SD**	3.64 ± 0.86		3.75 ± 0.89		0.113	0.2
How important do you think it is for students in the health professions to learn about planetary health?	Not important	-	-	-	-		
	Of little importance	2	2.2%	4	4.4%		
	Important	35	38.9%	24	26.7%		
	Very important	28	31.1%	31	34.4%		
	Extremely important	24	26.7%	30	33.3%		
	Non-respondents	1	1.1%	1	1.1%		
	**Mean ± SD**	3.83 ± 0.85		3.98 ± 0.89		0.074	0.24
How interconnected do you feel within the environment?	Not interconnected	-	-	-	-		
	Little interconnected	17	18.9%	13	14.4%		
	Interconnected	44	48.9%	36	40.0%		
	Very interconnected	21	23.3%	26	28.9%		
	Totally interconnected	8	8.9%	15	16.7%		
	Non-respondents	-	-	-	-		
	**Mean ± SD**	3.22 ± 0.85		3.48 ± 0.93		0.003*	0.46

Table S1. Pre and post-evaluation of perceptions about planetary health (n=90).

How important do you think it is for physicians to apply planetary health in their practice?	Not important	-	-	1	1.1%		
	Of little importance	3	3.3%	4	4.4%		
	Important	41	45.6%	26	28.9%		
	Very important	33	36.7%	29	32.2%		
	Extremely important	13	14.4%	30	33.3%		
	Non-respondents	-	-	-	-		
	**Mean ± SD**	3.62 ± 0.77		3.92 ± 0.95		0.002*	0.46
How much do you feel you learned in these classes? (post-test only)	Nothing	-	-	2	2.2%		
	Little	-	-	12	13.3%		
	Neither much, nor little	-	-	15	16.7%	-	-
	Much	-	-	43	47.8%		
	Extremely	-	-	17	18.9%		
	Non-respondents			1	1.1%		

*p<0.05

Table S2. Knowledge about diseases related to environmental changes (n=89).

Which of the following health problems in practice are associated with environmental changes, such as climate change? (select as many as you think convenient)					
**Disease group**		Pre**	Post**	p	r
**Chronic Non-communicable Diseases**	Asthma/Chronic Obstructive Pulmonary Disease	82 (91.1%)	88 (98.9%)	0.021*	0.75
	Cardiovascular Disease (stroke and acute myocardial infarction)	46 (51.7%)	82 (92.1%)	<0.001*	0.95
	Cancer	65 (73.0%)	78 (87.6%)	0.005*	0.52
	Diabetes Mellitus	34 (38.2%)	70 (78.7%)	<0.001*	0.95
	Systemic Arterial Hypertension	40 (44.9%)	77 (86.5%)	<0.001*	0.95
**Mental**	Depression	55 (61.8%)	77 (86.5%)	<0.001*	0.92
	Anxiety	64 (71.9%)	79 (88.8%)	<0.001*	0.88
	Post-Traumatic Stress Disorder	37 (41.6%)	66 (74.2%)	<0.001*	0.83
**Vector-borne**	Malaria	68 (76.4%)	82 (92.1%)	0.001*	0.78
	Dengue	73 (82.0%)	82 (92.1%)	0.004*	0.82
	Yellow Fever	68 (76.4%)	82 (92.1%)	<0.001*	1
**Heat-Related**	Dehydration	72 (80.9%)	84 (93.3%)	0.009*	0.52
	Hypotension	29 (32.6%)	66 (74.2%)	<0.001*	1
**Nutrition-Related**	Malnutrition	60 (67.4%)	78 (87.6%)	0.001*	0.6
	Obesity	40 (44.9%)	77 (86.5%)	<0.001*	0.9
**Respiratory acute disease**	Pneumonia	63 (70.8%)	82 (92.1%)	<0.001*	0.83

*p<0.05 ; **Categorical values were described as n (%).

Table S3. General Methodology of PH Meetings

“What did you think of the teaching methodology used?”

Class 1	n	Examples
General positive feedback	20	"Very well constructed", "effective", and "accurate".
Praise for active participation, creativity, and interactivity	7	"I believe it's intuitive and creative.", "Very interactive", "I felt free to think about the subject I was going to approach, without great demands from the audience.", and "the students were the protagonists of the learning process."
Praise for the final presentations	6	"I found the portfolio and group presentation productive! It wasn't burdensome for anyone, and everyone could delve into their part without losing the bigger picture."
Praise for the real patient interviews	3	"I found the proposal to search for a real patient and then make a very good presentation."
Praise for the provided references	2	"The PDF with reference suggestions also greatly helped in using reliable and informative sources."
General criticism	1	"Excessively playful."
Praise for the portfolio	1	"I found the portfolio productive."
**Class 2**	**n**	
General positive feedback	25	"Quite didactic", "provided a lot of learning to all of us", and "All the activities were very integrated and complementary."
Criticism of lecture	16	"Of little relevance and extremely tiring, a subject for just one interactive class and not a video that could be watched at home at an accelerated speed."
Praise for the real patient interviews	8	"It was very interesting to try to understand the environment in which he [the patient] is inserted."
General criticism	6	"I would have liked to have contact with more technical knowledge and less playful.", "Too long", "heavy", and "the topic was approached in a very superficial way."
Praise for active participation, creativity, and interactivity	3	"I found it creative", "very participatory", and "the active methodology helps in understanding the addressed content."
Praise for the real patient interviews	3	"Constructing the presentation was a good moment for the group."
Criticism of real patient interviews	3	"Choosing the patient (...) caused difficulty (...) in contextualizing the topic", "It seems like a lack of sensitivity to ask about heatwaves (...) after the patient reported having had very serious illnesses."
Praise for the provided references	3	"Support material (very complete)."
Praise for the portfolio	2	"I believe the most productive part was (...) the organization of the portfolio."

Table S4. Impact on Students' Personal Lives - Classes 1 and 2

"How can the meetings on planetary health help you in your professional practice?"

Category	n	Examples
Understanding, reflection, and awareness.	26	"Understanding more about the impacts that climate change can generate in different areas." "I have gained a better understanding of the importance of environmental preservation for my own health." "They have prompted reflections on everyday practices that can be more beneficial to me and to the whole." "Raising awareness about our role in environmental preservation."
Change of personal lifestyle habits	25	"Observing my surroundings and readjusting my actions in order to reduce ecological footprints, as well as avoiding contact with risk factors."
Valuing your well-being in connection with the environment.	7	"Self-care", "appreciating a deeper connection with nature that used to seem like senseless bucolism, now I can see meaning behind it", and "realizing that connecting with nature is not a privilege but a necessity and a right."
Implementing system change.	4	"Being actively involved in the fight against environmental issues" and "having awareness in leadership roles, if occupied."
Assists in your life in an unspecified way	2	"Yes, very much" and "In a significant way."
Assists due to engaging in debate	1	"The exercise of tolerance and debate of ideas is fundamental for the development of any person and was greatly practiced during the meetings. In addition, the contact with diverse opinions about planetary health contributed to expanding the discussion already proposed in the lecture."
Talking about the subject with others	1	"They help me to expose how climate change impacts people's lives."
Eco-anxiety	1	"Adding to the anxiety about environmental degradation."
No (new) applications	4	"(...) It doesn't have much applicability since we can't avoid certain exposures." and "Not much, since I am very concerned about environmental well-being."

Table S5. Impact on Professional Practice of Students - Classes 1 and 2

"How can the meetings on planetary health help you in your personal life?"

Class 1	n	Examples
They allowed for an expansion of the perspective on health and illness and an understanding of diseases and PH.	41	"Better understanding the etiology of certain diseases, considering that imbalances in the planet's 'homeostasis' cause illnesses." "The context of life and diseases may be even more interconnected than I thought." "Investigating the patient's engagement with the environment around them can reveal a lot about their health." "They can enhance the understanding of the situations that permeate the patient's life, from their pathologies to their social, economic, cultural, etc., context."
They enabled a better approach with the patient in order to encourage them to protect themselves from risk factors and to change their habits.	29	"Treating the patient within their context and not just focusing on the disease." "Guiding patients and suggesting lifestyle changes." "Striving to impact my patients by influencing them to better care for the environment." "Simultaneously, considering alternative treatments that involve addressing potential environmental issues." "Alerting patients about the risks of exposures."
They assisted in a nonspecific manner.	7	“Promoting a more sustainable daily conduct." "The sharing of experiences and learning from an experienced professional primarily aided in the application and contextualization of the topic."
They sparked an interest in studying this topic.	5	"To become more attentive to the subject and seek a better understanding of it" and "to seek information and further training."
They encouraged reducing the environmental footprint of the healthcare system.	4	"Learning to prevent waste, such as excessive tests and medications." "Assisting in the process toward a more energy-efficient and less polluting healthcare system." "They can help me by enabling me to provide guidance within the hospital setting."
They contributed to them becoming better doctors.	3	"They can make us better primary care physicians."
Little application	2	"I don't believe it's so relevant to the field." "If they bring me solutions and ideas applicable to our context, I think it could add..."
System change.	1	"Expand one's horizons beyond the patient, aiming to act not only as a physician but also as a guide for the population."”

Student Suggestions

Responses to the question: "Your observations about these planetary health meetings and suggestions to improve it".

Suggestions from Class 1:

1. An increased number of meetings to allow a deeper understanding of the topic (n=8). Comments included, "I believe there could be smaller group meetings to discuss the complexity of the topics." and "The meetings could span a longer period, encompassing the entire Integrative course. With each portfolio, there should be a prompt about the SP approach. The goal of the integrator course aligns well with the topic.";
2. More recommendations for practical application (n=5). Comments were, "Physician's actions with practical examples.", "Discuss more about what can be done on the topic.", and "Real projects for HCPA (university hospital).";
3. Greater support for preparing patient interviews (n=2);
4. More assistance in preparing presentations (n=1);
5. Reduced class time dedicated to SP (n=1);
6. One student suggested an elective course on the subject, while another felt that a course on this theme might not be a good idea, stating, "What becomes obligatory becomes burdensome.";
7. Nine students requested a theoretical lecture on the topic. Comments were, "I believe there should be a theoretical class to refine the concepts: first, students understand the terms and then present on them." and "If it started with an introductory class, like Dr. Rafaela's video for UFCSPA, I believe it would spark more interest in the students."
8. Three students commented on the tumultuous academic period during which the classes took place and made suggestions: "The activity occurred during a very tumultuous academic period (full of exams). I believe improvements can be made by valuing the class time and setting aside a week without in-person activity for project development.",

“They found it difficult to balance with other school obligations.” and “I believe it should be done at the beginning of the semester when students are not so overwhelmed with exams. Also, I think it would be better addressed in the second semester, as the third has a large workload and is characterized as the most challenging of the course.”

Suggestions from Class 2:

1. Twelve students suggested expanding the sessions on Planetary Health (SP) and delving deeper into the topic: “I felt it was very much on the lines of intrinsic general knowledge.”, “The time limitation prevented many subjects from being explored in deserved depth.”, “More time between tasks would aid in better presentation and portfolio preparation.”, “The content and activities were too heavy for just three classes.” and “Greater theoretical depth: understanding the physiological effects of pollutants, pesticides, etc. on our bodies would be great!”

   Highlighting the aspect of depth: "We need to learn a lesson: recognizing that environmental protection is a lost cause if we don't find incentives for the general public, not just their representatives, to defend it. As constructive feedback, I believe the approach of this module is ill-suited for medical school. It borders on a perspective more suitable for social sciences and direct environmental professions. The current tone of fostering a naive, utopian love for the environment won't achieve the desired impact. Other topics like epigenetics and genotoxicity related to microplastic exposure, understanding clinical outcomes based on particulate nature would be more interesting and less generalized than the current approach.”;
2. One student suggested creating an elective course, one was against an elective and in favor of keeping it within the integrative course, and another suggested the topic be covered in other courses;

3. Seven students suggested reducing the class hours for SP, mentioning an overload of out-of-class tasks. Suggestions ranged from having only one lecture, only one participatory class, a lecture + discussion, lecture + patient interview, patient interview + discussion, or a lecture + photo discussion. One student suggested selecting a more "suitable" patient;
4. Regarding the theoretical lecture, suggestions (n=11) included: having it in-person and live, allowing instant interaction/debate with professors, letting students watch it at home, dividing the video into 2 or 3 parts, and providing more technical content. Additionally, one student mentioned integrating visual productions like documentaries or films on the topic would be welcome;
5. Concerning the timing of sessions, nine students commented that the semester they are in isn't ideal and/or that the coinciding exams from other courses significantly impacted their study and engagement: Hold it in the first month of classes, so it doesn't coincide with exam periods for better engagement. Comments included, "The activities demanded a lot during a semester that is already heavy.", "It adds more workload to an already challenging semester.", "Suggestion: different semester.", "Heavy methodology in an already demanding semester; suggest covering this topic in Integrative II." and "It might be interesting to address this theme in the first semester."
